# A hierarchical clustering method of hydrogen bond networks in liquid water undergoing shear flow

**DOI:** 10.1038/s41598-021-88810-7

**Published:** 2021-05-05

**Authors:** Yitian Gao, Hongwei Fang, Ke Ni

**Affiliations:** State Key Laboratory of Hydro-Science and Engineering, Department of Hydraulic Engineering, Tsinghua University, Beijing, 100084 China

**Keywords:** Chemical physics, Thermodynamics

## Abstract

Many properties of water, such as turbulent flow, are closely related to water clusters, whereas how water clusters form and transform in bulk water remains unclear. A hierarchical clustering method is introduced to search out water clusters in hydrogen bonded network based on modified Louvain algorithm of graph community. Hydrogen bonds, rings and fragments are considered as 1st-, 2nd-, and 3rd-level structures, respectively. The distribution, dynamics and structural characteristics of 4th- and 5th-level clusters undergoing non-shear- and shear-driven flow are also analyzed at various temperatures. At low temperatures, nearly 50% of water molecules are included in clusters. Over 60% of clusters remain unchanged between neighboring configurations. Obvious collective translational motion of clusters is observed. The topological difference for clusters is elucidated between the inner layer, which favors 6-membered rings, and the external surface layer, which contains more 5-membered rings. Temperature and shearing can not only accelerate the transformation or destruction of clusters at all levels but also change cluster structures. The assembly of large clusters can be used to discretize continuous liquid water to elucidate the properties of liquid water.

## Introduction

Water is the most ubiquitous substance on Earth and is the key solvent in almost all chemical, biological and geological processes. Despite high abundance, water has various anomalous physical–chemical properties, such as a density maximum at 4 °C, the steep increase in the isothermal compressibility and heat capacity upon cooling, the non-Arrhenius behavior of viscosity and diffusion constant at low pressures and many more^1,2^. It is universally acknowledged that many anomalous properties of water are related to its peculiar microscopic structure, which has remained a mystery over the decades^3^. Water molecules are connected with each other by hydrogen bonds to form a three-dimensional network structure^4^. Inside the structure of water, a subtle balance is spontaneously formed between directional hydrogen-bonded interactions and nondirectional weaker van der Waals interactions^5^, which can perfectly explain water anomalies. Accordingly, it is critical to explore the microscopic structure of liquid water.

According to many spectral experiments^6,7^ and molecular simulations^8,9^, a single water molecule forms tetrahedral hydrogen bonds with its surrounding water molecules involving distorted configurations. However, Wernet et al*.*^10^ concluded that the “ring-and-chain”-like structure is favored in water based on X-ray absorption and Raman scattering experiments, and a topic of intense debate was raised as to whether water is tetrahedral or “ring-and-chain”-like in structure^11^. It is universally acknowledged that liquid water is considered as random three-dimensional hydrogen bond network continually undergoing topological reformation^12,13^. Based on Raman spectroscopy^7^, X-ray emission spectroscopy^14^ and small-angle X-ray scattering spectroscopy^15^, the results^16–20^ suggested that tetrahedral low-density liquid (LDL) and distorted high-density liquid (HDL) structures coexist in heterogeneous networks.

Water clusters including hundreds of water molecules have been exhaustively investigated using experiments and ab initio molecular simulations^13,21,22^. However, hydrogen bonds between water molecules are in dynamic equilibrium and frequent breaking and reforming of hydrogen bonds are occurred cooperatively within water clusters. Water clusters are short-life and flickering which life spans are estimated from 10^−10^ to 10^−11^ s^23,24^. Therefore, it remains a mystery how flickering water clusters form bulk water^25^. Macroscopically, Navier–Stokes equation can describe the motion of fluid particles in bulk water, which can be regarded as giant water clusters. Turbulence in a fluid flow is characterized by irregular and chaotic motion of fluid particles, whose velocities change rapidly in space and time. In turbulent shear flow, the eddies forming the fluid particles generate and then degenerate after traveling a certain distance to change their momentum by a new environment in the fluid^26^. Viscosity arises from the exchange of fluid particles between different velocity shear layers. Turbulent also causes an increased rate of momentum transfer, which essentially includes low momentum diffusion (the mixing of mass without bulk motion), high momentum advection (the mixing of mass with bulk motion)^26^. Accordingly, the characteristics of water clusters related to bulk motion can elucidate turbulent diffusivity^26^. To form a bridge between macroscopic hydraulics and microscopic molecular dynamics, a bottom-up approach of searching out water clusters by molecular dynamics is necessary to explore detailed structure and dynamics of water clusters.

Graph theory is a branch of mathematics that studies the topology of graphs, and it has been successfully applied to biological, informational and economic systems. Many investigations^27–34^ have introduced the concept of graph theory to explore topological properties of hydrogen bond networks in liquid water. The topological characteristics of the global network are easily analyzed by calculating clustering coefficient, graph spectra, etc^27,33^. As a cyclic path connected by hydrogen bonds, rings mostly cover the global hydrogen bond network^30^. Unlike ice Ih containing only perfect 6-membered rings, liquid water shows a broader ring size distribution upon heating due to more intense thermal motion^27–29^. In addition, 4-, 5- and 6-membered rings are fundamental structural units in larger water clusters based on ab initio calculations^35^ and molecular dynamic simulations^36^. Regarding rings as building units, fragments have been constructed by Matsumoto as three-dimensional units of network^31^. Fragments mutually aggregate to construct stable aggregates^31^. However, few attempts have been made to explore more complex water cluster structures in bulk water based on molecular dynamic simulations. Furthermore, the traditional definition of clusters using graph theory gives a poor perspective of the hydrogen bond network in water. Two molecules are regarded as belonging to the same cluster if they are connected by a chain of hydrogen bonds, as a result of which large clusters probably percolate throughout the system instead of isolated water clusters^37^. It is necessary to propose a reasonable definition to partition the hydrogen bond network in liquid water and attain a set of large water clusters for a discrete description of water.

In this paper, we aim to develop a graph-based hierarchical clustering method to provide a comprehensive overview of water clusters at all levels in liquid water undergoing both non-shear- and shear-driven flow. Hydrogen bonds are regarded as *first-level structures.* 4-, 5- and 6-membered rings, regarded as *second-level structures*, constitute primary building units of fragments, regarded as *third-level structures*. Using the modified Louvain algorithm of the graph community, high-level water clusters are merged by low-level water clusters based on indirect graphs of low-level water clusters. All sets of clusters at different levels constitute hierarchical clustering lists after repeating agglomeration. Molecular dynamic simulations are carried out under different temperatures and shear-driven flow to study the properties of hierarchical water clusters and the quantitative distribution, cluster transformation, cluster lifetime, etc. are further analyzed.

## Results

### Molecular dynamics simulations and hierarchical graph-based clustering method

Molecular dynamics simulations are performed using periodic boundary conditions with 17,314 water molecules in a cubic box interacting through SPC/E intermolecular potential^38^. The long-range electrostatic interactions were calculated with particle–particle particle-mesh solver (PPPM) summation. All of the simulations were run at temperatures of 240 K, 260 K, 280 K, and 300 K and a pressure of 1.0 atm with a Nose–Hoover thermostat and barostat. The time step was 1.0 fs. The systems were firstly equilibrated for 2.0 ns in an NVT ensemble. This was followed by a NPT simulation for 10.0 ns. Then, the third period of 7.0 ns was used to attain shearing flow using SLLOD algorithm^39–41^.

Similar to cluster, graph community is a structure in which the nodes gather into groups and have a higher density of connections within groups than between them. Louvain algorithm^42^ is broadly used for community detection to unfolds a complete hierarchical community structure for network according to high *modularity* partitions. Modified Louvain algorithm proposed in this paper takes *adjusted mutual information* into consideration for higher similarity of clustering between neighbor configurations to attain stable clustering partitions. If clusters, regarded as vertices, have shared molecules, there exists an edge between clusters, the weights of which are the number of shared molecules. Based on modified Louvain algorithm, the network of lower-level clusters can search for community regarded as higher-level clusters. The hierarchical clustering method is used to attain a dendrogram of clusters at different levels by successively merging lower-level clusters to form higher-level large clusters.

### Hierarchical cluster analysis

This section introduces the definition of hierarchical water clusters. Hydrogen bonds, rings and fragments are considered 1st-, 2nd-, and 3rd-level structures. The networks of fragments are regarded as starting conditions to search for water clusters at all levels successively by modified Louvain algorithm.

#### First-level structure: hydrogen bond

**Figure 1:**
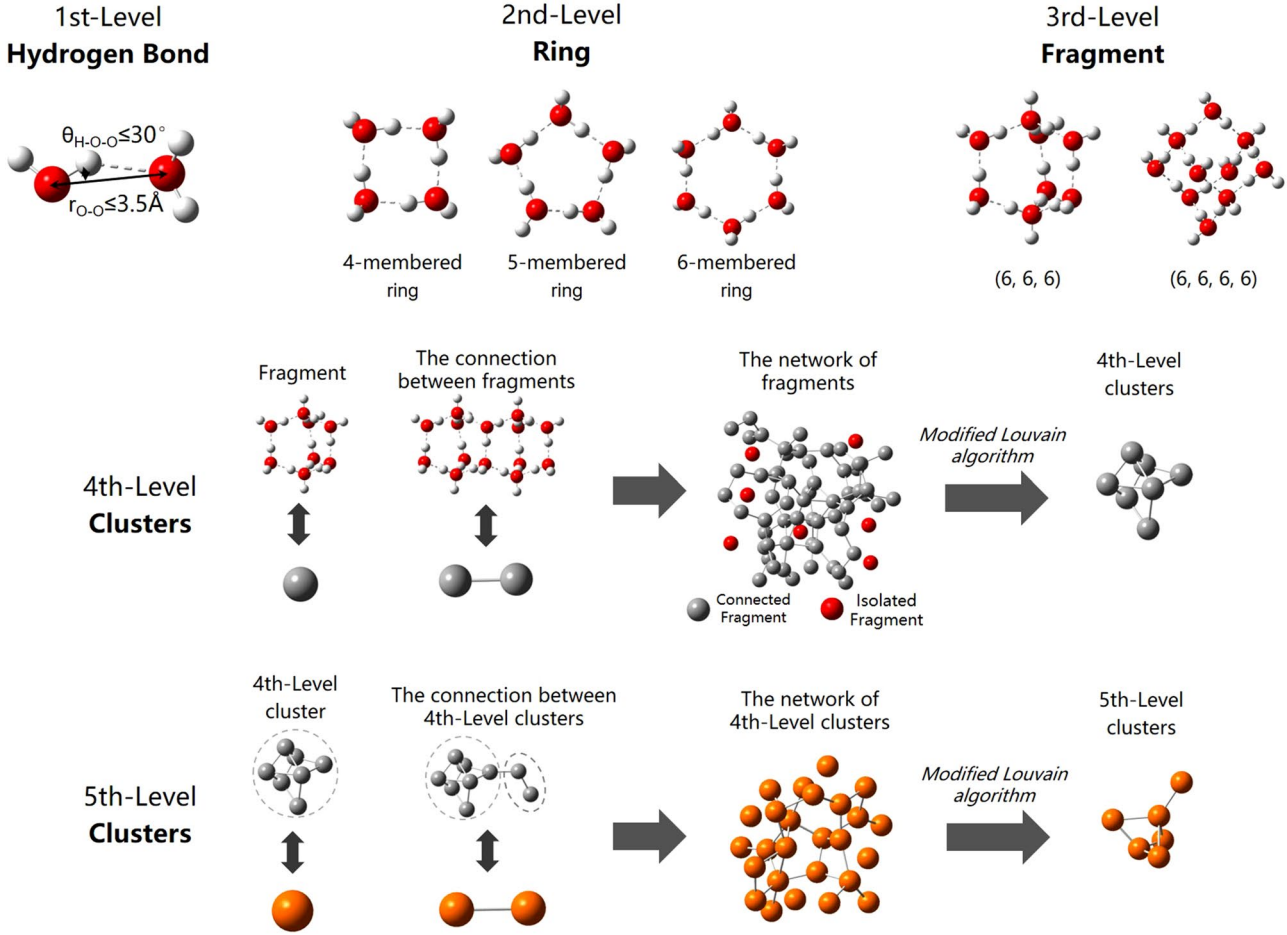
Schematics of hierarchical clustering method proposed in this study. Hydrogen bonds, rings and fragments are considered as 1st-, 2nd-, and 3rd-level structures which is ball-and-stick models from a chemical perspective and the red and white balls denote oxygen and hydrogen atoms, respectively. The full and dotted sticks denote O–H covalent bonds and hydrogen bonds, respectively. The 4th- and 5th-level clusters are illustrated by topological perspective. The balls represents the structure of last levels. Note that the structures in the figure are only a selection among the considered ones by the clustering algorithm. The structures are drawn by GaussView 5.0.8^50^.

Hydrogen bonds are defined as the first-level structures of water network. It has been established that geometric criteria have better performance in reproducing hydrogen bond rearrangements and network topology of inherent structure^43^. Therefore, we choose a popular geometric standard in which two water molecules are regarded as hydrogen bonded when the distance between two oxygen atoms is less than 0.35 nm and the hydrogen–oxygen-oxygen angle is less than 30°^44^, as shown in Fig. 1. The direction of the hydrogen bonds was neglected for simplicity to consider water network as an undirected graph^31^.

#### Second-level structure: ring

Rings are defined as the second-level structures of water network. In Fig. 1, the primitive ring is a cyclic path along the “edge”, which can not be decomposed into smaller rings. The "shortest-path" (SP) criterion developed by Franzblau et al.^45^ was utilized for ring statistical analysis.

In the global hydrogen bonded network, 4–8-membered rings predominate^30^. 6-membered rings are the most popular type at ambient temperatures. The average lifetimes of the 5-membered rings (458.4 fs) are longer than 6-membered (399.4 fs), 4-membered (294.6 fs) and 7-membered (288.5 fs) at 240 K. According to molecular dynamics stimulation^36^ and ab initio stimulation^35^, low-energy water clusters (*n* ≤ 20) mainly consist of 4-, 5-, and 6-membered rings. The optimal structure of clusters strikes a balance between tetrahedral coordination which favors 6-membered rings and the structure of eliminating surface dangling atoms and increasing the number of hydrogen bonds which favors smaller rings^36^. Accordingly, to search for stable and low-energy clusters, 4-, 5- and 6-membered rings are considered as basic building rings.

#### Third-level structure: fragment

Fragments, proposed by Matsumoto^31^, are a type of cagelike structures covered by rings satisfying topological conditions (Detailed conditions can refer to Ref.^31^). Fragments broadly distribute in hydrogen-bonded network of water in a tessellated fashion at low temperature^31^.

In this study, all rings in fragments must be 4-, 5-, 6-membered rings to search for stable clusters. It has been investigated that over 50% fragments only have 3 and 4 rings and the fragments constituted by more rings may not be compacted for a big hole inside the cage^31^. Therefore, it is assumed that the number of rings in fragments must be less than 4. Similar to the destruction of hydrogen bonds, the destruction of fragments might involve two methods: temporary destruction and genuine destruction^46–48^. For more stable structures, we neglect transient destruction of fragments. The maximum permissible temporary destruction of the fragment is 100 fs during which the fragments are recombined together after several hydrogen bonds are broken transiently and the temporary destruction is considered to be a part of its total lifetime. Approximately 85.0% fragments have the non-lifetimes less than 100 fs. The detailed information is intrdoced in Supplementary Figure S1.

#### N(N > 3)th-level structure: clusters

A water cluster is an assembly of weakly bound water molecules, and there are numerous local minima on the potential-energy hypersurface of water clusters based on the results of quantum chemical calculations^49^. In most cases, the more hydrogen bonds the clusters include, the lower potential energy the clusters may have. Traditionally, two molecules belong to the same cluster if they are connected by a continuous path of hydrogen bonds^37^, which forms percolated clusters spanning the periodic cubic simulation box at least in one direction at ambient temperatures instead of isolated clusters.

Different from traditional definition, communities are patches that have denser connections with each other via edges. The networks of fragments are regarded as starting conditions. Modified Louvain algorithm is proposed to search for water clusters successively. In this paper, the maximum level of hierarchical clusters is 5.

### Radial distribution function and hydrogen bond network

**Figure 2:**
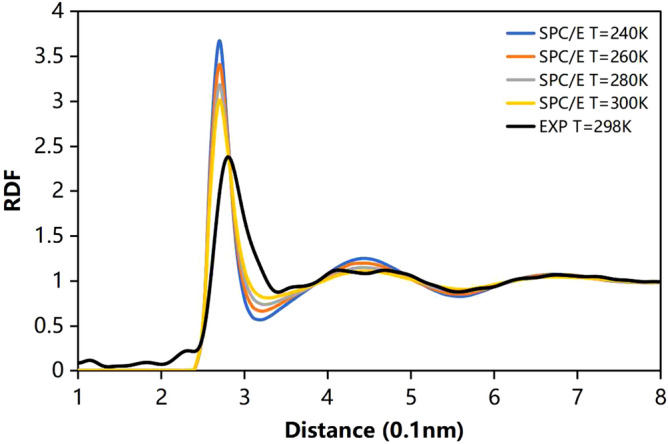
The radial distribution function (RDF) of oxygen–oxygen at various temperatures.

The calculated macroscopic properties are in agreement with previous simulation results^51–55^ (Supplementary Table S1). In Fig. 2, we plot radial distribution functions for oxygen–oxygen (O–O). Compared with the results from X-ray scattering^56^ and neutron-scattering experiments^57^, water shows similar structural characteristics. The first peak represents the first shell at about 2.8 Å and the second shell at about 4.5 Å, whereas a trough between 3 and 4 Å indicates interstitial water. As temperature increases, the first and second peaks distinctly flatten because of the destruction of tetrahedral structure for stronger thermal fluctuation. The curves of H–O and H–H RDF have similar rules in Supplementary Figure S2. These results are in good agreement with other simulations^58–60^.

### Hierarchical cluster distribution

The distributions of hydrogen bonds, rings, fragments are compared with previous literatures for verification. The distributions of 4th- and 5th-level clusters are also discussed under the influences of temperature, shearing and clustering levels.

#### First-level structures: hydrogen bonds

**Figure 3:**
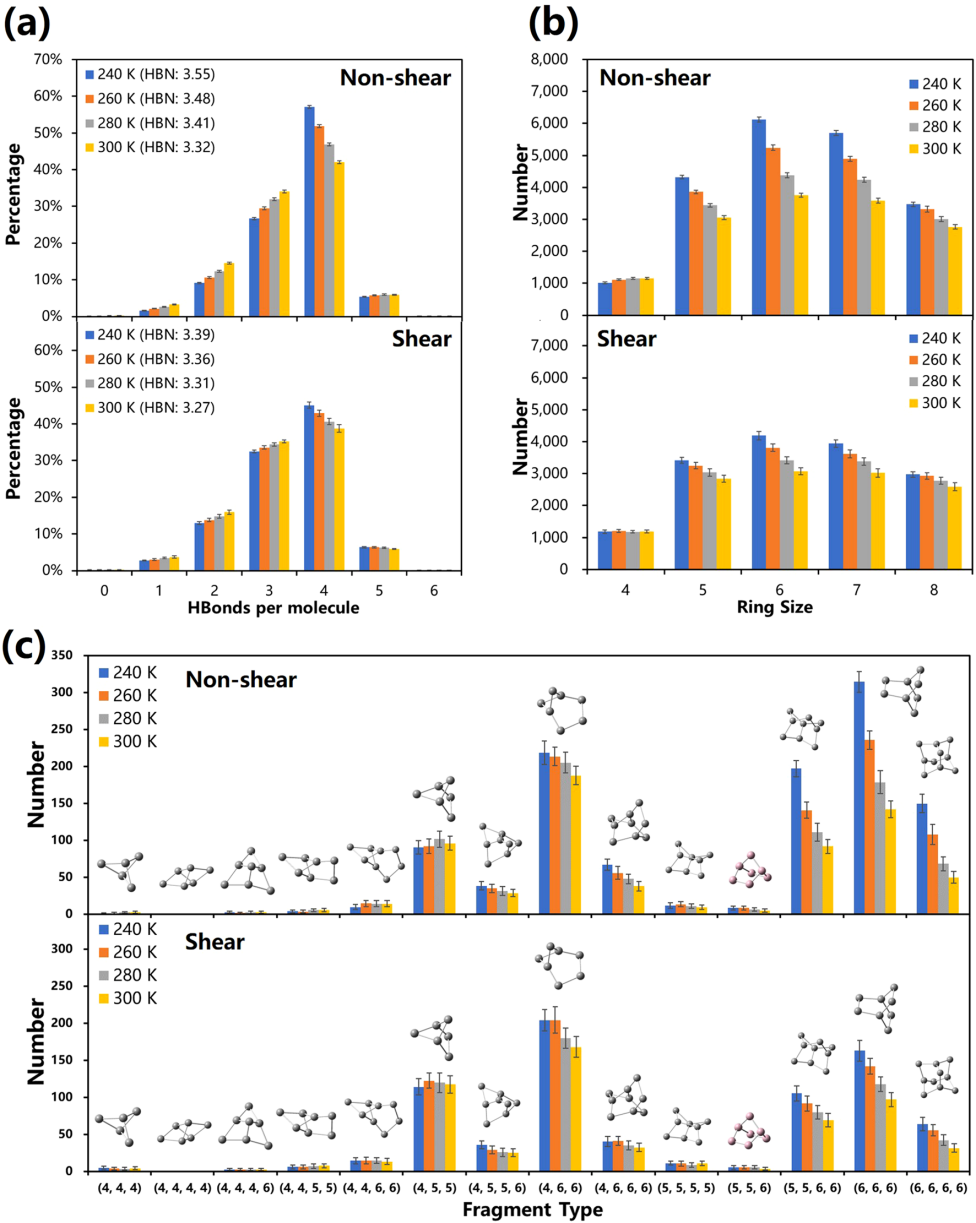
The distribution of hierarchical structures at the 1st, 2nd and 3rd levels in the network in various cases. (**a**) The distribution of hydrogen bonds (1st-level structures) at various temperatures. (**b**) The distribution of rings (2nd-level structures) at various temperatures. (**c**) The distribution of fragments (3rd-level structures) at various temperatures. Note that (4, 4, 4) denotes a symbol of a fragment including three 4-membered rings. The structures are drawn by GaussView 5.0.8^50^.

In Fig. 3a, 67.40%, 61.04%, 54.56% and 49.56% of water molecules are respectively coordinated to form perfect tetrahedral structures at 240 K, 260 K, 280 K and 300 K in non-shear cases, showing a decrease upon heating. The number of 2-, 3- and 5-coordinated water molecules produced from broken tetrahedral structures increase upon heating. Shear-driven flow destroys 4-coordinated molecules, similar to the effect of heating. At 240 K, the percentage of 4-coordinated molecules in shear-driven flow is 11% less than in non-shear flow, equivalent to heating up to 30 K. As temperature increases, average number of hydrogen bonds per a molecule decreases monotonically from 3.764 (non-shear) and 3.590 (shear) at 240 K to 3.524 (non-shear) and 3.420 (shear) at 300 K. Compared with non-shear flow, average number of hydrogen bonds in shear-driven flow obviously decreases by 5%. In general, both temperature and shearing can tremendously destroy hydrogen network.

#### Second-level structures: rings

In Fig. 3b, 5-, 6-, and 7-membered rings are superior in numbers in all simulated cases. Six-membered rings are mostly favored, which constitute characteristic cage-like structures in ice *Ih*. With increasement of temperature, 5-, 6-, 7-, and 8-membered rings gradually reduce and smaller rings slightly increase, which confirms that heating can damage tetrahedral structures. The decreasing of 5-, 6-, and 7-membered rings under shearing proves that the ordered motion generated by tvelocity gradient may cursh large rings into small ones. Since SLLOD algorithm sets a homogeneous thermostat on global system to attain a steady state^39–41^, it might neglect the effect of shear heat.

#### Third-level structures: fragments

As three-dimensional building blocks, fragments tessellate in hydrogen-bonded network, 41.1% of which are covered by fragments in non-shear-driven flow at 240 K and 31.6% in shear-driven flow. However, at 300 K, only 27.2% and 22.9% of network is covered by fragments in both cases. Entangled networks are intersitial between water clusters. In Fig. 3c, fragments are classified into two types^31^: cryophile type, whose number decreases upon heating, and thermophile type, whose number increases upon heating. The fragments mainly including 5-, 6-membered rings have comparatively larger numbers than ones including 4-membered rings. The 4-membered rings cause extremely great distortion of fragments that have much shorter lifetimes. The composite patterns of fragments which have smaller distortions are favored, such as (4, 6, 6), (5, 5, 6, 6), (6, 6, 6) and (6, 6, 6, 6). Due to the reduction of 5-, 6-membered rings, the number of fragments including 5-, 6-membered rings almost decreases with heating. In shear-driven flow, the number of almost all fragments decreases because of decreasing number of rings.

#### Nth(N > 3)-level structures: clusters

**Figure 4:**
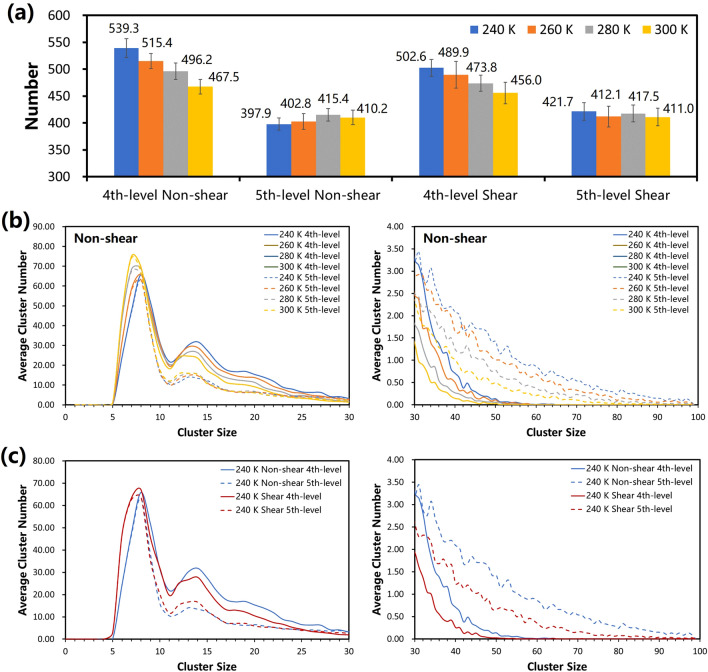
The distribution of hierarchical structures at the 4th and 5th levels in the network in various cases. (**a**) The average number of 4th and 5th-level clusters at various temperatures. (**b**) The average distribution of 4th- and 5th-level cluster sizes at various temperatures.

In Fig. 4a, with the increasement of temperature or the effect of shearing, average number of 4th-level clusters have an obvious trendency towards decreasing, whereas average number of 5th-level clusters do not have significant regular variation. In Fig. 4b, after the operation of level-up by hierarchical clustering method, the number of 5th-level clusters including 8–30 molecules is evidently decreasing compared with the number of 4th-level clusters, whereas over-30-molecules 5th-level clusters are merged more. It is indicated that the number of 5th-level clusters is less than 4th-level because of agglomeration of level-up. With the comparsion of deviation at different temperatures, higher temperature corresponds to weaker agglomeration ability. The reason is that at high temperature the clusters are reduced in number and arranged loosly and there are less probability of merging into larger ones. The ambiguous variation of 5th-level clusters is concerned with contradictory mechanism between quantitative change of clusters at higher temperatures and high levels.

In Fig. 4c, similar to heating up, shearing can decrease the number of 4th-level clusters including more than 10 molecules because of the decrease of fragments. However, the number of 5th-level clusters shows similar rules that 30-molecules 5th-level clusters are a critical point of the change of cluster number.

**Figure 5:**
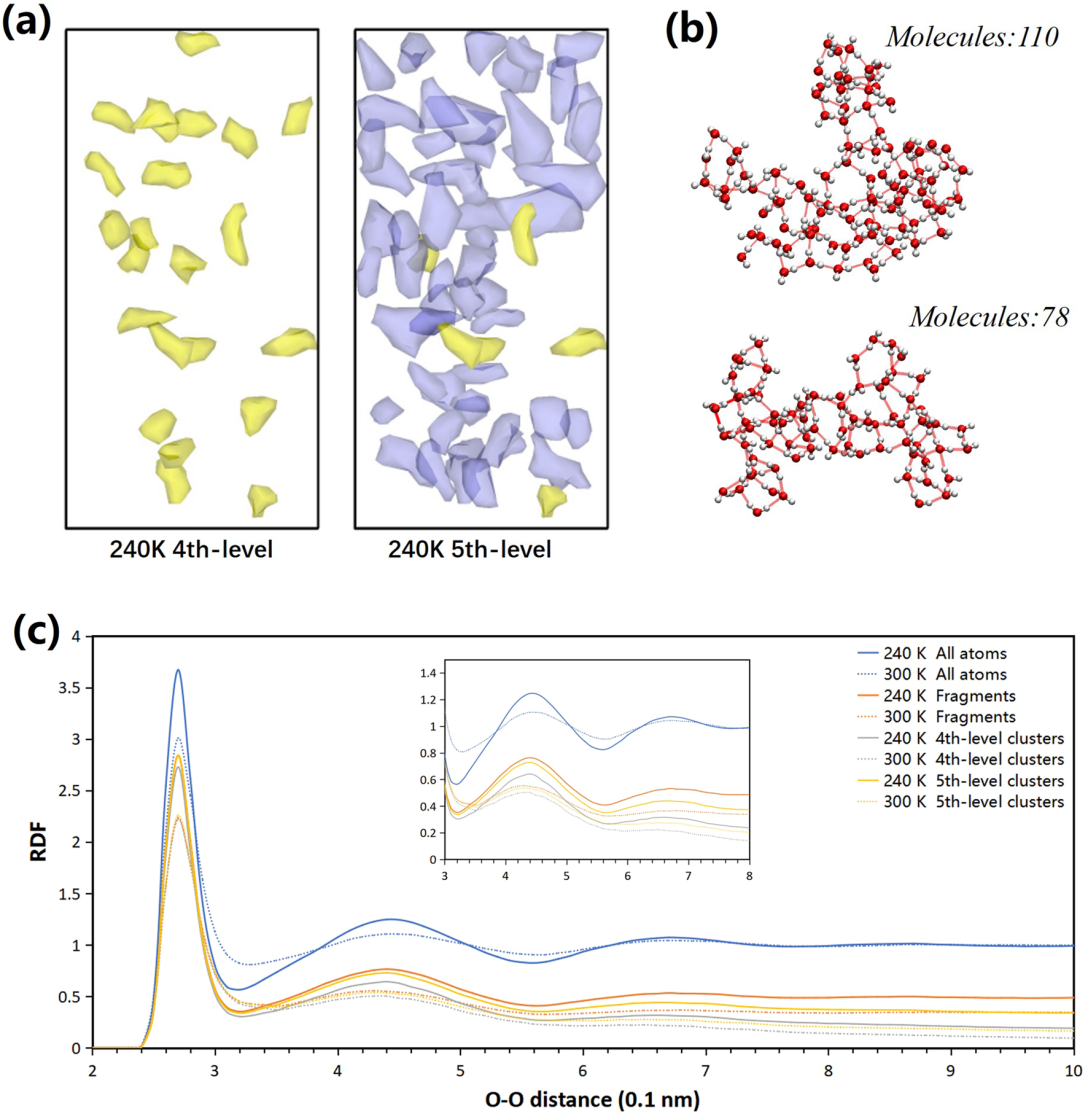
The spatial distribution of the 4th- and 5th-level clusters at 240 K and radial distance functions of the 4th- and 5th-level clusters at 240 K and 300 K. (**a**) 4th- and 5th-level large clusters that have more than 30 molecules at 240 K drawn by Ovito^62^. (**b**) The ball-and-stick model of characteristic clusters drawn by VMD 1.9.1^63^. (**c**) The radial distance functions of the 4th- and 5th-level clusters at 240 K and 300 K. The radial distance functions are calculated in the selected oxygen atoms.

As shown in Fig. 5a, we can compare spatial distribution of 4th- and 5th-level clusters at 240 K. The clusters at both levels are surrounded by interstitial water molecules which do not belong to any clusters. At 4th level, large clusters that have more than 30 molecules are randomly scattered in the box. However, based on 4th-level clusters, 5th-level clusters have distinct agglomeration of small clusters and have a higher proportion of large clusters. Under different temperatures, low temperature aggregates more large clusters. At 240 K, only 15–17% of the 4th-level clusters are classified as large clusters including more than 30 molecules and more than 25% of 5th-level clusters are classified as large clusters, which include approximately 25% of the water molecules. In Fig. 5b, we illustrate the ball-and-stick model of characteristic clusters. The average maximum cluster size at both levels in non-shear-driven flow shrinks from 49.7 (4th level) and 112.1 (5th level) at 240 K to 41.0 (4th level) and 73.8 (5th level) at 300 K. When the temperature is lower than 260 K, average maximum cluster size at the 5th level exceeds 100 molecules. Upon shearing, the average maximum cluster size also shrinks from 43.0 (4th level) and 85.8 (5th level) at 240 K to 39.0 (4th level) and 70.4 (5th level) at 300 K.

In Fig. 5c, we illustrate radial distance functions of selected oxygen atoms^61^. We choose four cases: all oxygen atoms, oxygen atoms belonging to fragments, oxygen atoms belonging to 4th-level clusters and oxygen atoms belonging to 5th-level clusters. The RDF of all oxygen atoms have peaks at 2.85 Å, 4.4 Å and 6.6 Å which indicates intrinsic tetrahedral structure. Similar to the RDF of all oxygen atoms, the RDF of other cases have obvious peaks at same places, which indicates that clusters retain tetrahedral structure. The RDF of 4th-, 5th-level clusters almost smooth the peaks at more than 6 Å, which may means maximum length of clusters. Besides, higher temperature cause the curve flattening at some peaks in all cases, especially higher level structures.

### Dynamics of hierarchical clusters

*AMI* (adjusted mutual information)^64^ is used to quantify the similarity of clustering in modified Louvain algorithm. Increasing temperature and shearing can cause *AMI* value monotonically declining, which indicates that stronger thermal fluctuation stimulates faster variations in clusters. Higher level of hierarchical clustering method corresponds to more unstable clustering. (Supplementary Table S2).

Cluster transformation has 4 patterns: (1) One cluster remains unchanged. (2) One cluster is totally merged into another cluster. (3) one cluster is split from another cluster. (4) One cluster is transformed into another cluster by complex fusion and fission. In non-shear-driven flow, the majority of clusters (76.6% at 240 K; 72.1% at 260 K; 69.0% at 280 K; 66.1% at 300 K) at 4th levels remain unchanged at various temperatures. The porportion of unchange clusters declines with heating up for stronger thermal fluctuations. At 5th level, the unchange rates (73.2% at 240 K; 69.2% at 260 K; 67.2% at 280 K; 64.8% at 300 K) slightly reduce for the unstability of large clusters. A certain number of clusters (approximately 10–15%) are totally merged into clusters or split into pieces, the number of which decreases with heating. A very small number of clusters (approximately 2–3%) have complex changes, most of which are large clusters. Compared with non-shear-driven flow, shearing leads to more stable clusters at both levels at low temperatures, whereas clusters have a tendency towards stability at high temperatures. The detail is reported in Supplementary Table S3.

At 240 K in non-shear-driven flow, average lifetime *t*_1_ of all clusters^65^ at the 4th and 5th levels (150.6 fs at 4th-level; 158.07 fs at 5th-level) is nearly as long as the lifetime of fragments, ~ 150 fs. Clusters only consisting of one or two fragments may have a longer lifetime. To eliminate the influence of clusters including only one fragment, the average lifetimes *t*_2_ of clusters means the lifetime of clustering excluding one-fragment clusters. Average lifetimes *t*_2_ of clusters at both levels (110.8 fs at 4th-level; 93.9 fs at 5th-level) obviously diminish. A greater proportion of single-fragment 5th-level clusters is the main reason why they have larger *t*_1_ but smaller *t*_2_. The large clusters are generally constituted by more than 5 fragments, equal to approximately 30 molecules. The average lifetimes *t*_3_ for large clusters at both levels (59.6 fs at 4th-level; 44.8 fs at 5th-level) are muchs shorter than *t*_2_, indicating that large clusters transform very rapidly and frequently. In shear-driven flow, the average lifetimes *t*_1_ are approximately 87.3% of that in non-shear-driven flow. The average lifetimes *t*_3_ of large clusters at the 4th and 5th levels are only 52.1 fs and 40.3 fs. The significantly shorter lifetime of clusters proves that heating and shearing can accelerate the destruction of clusters. The detail is reported in Supplementary Table S4.

**Figure 6:**
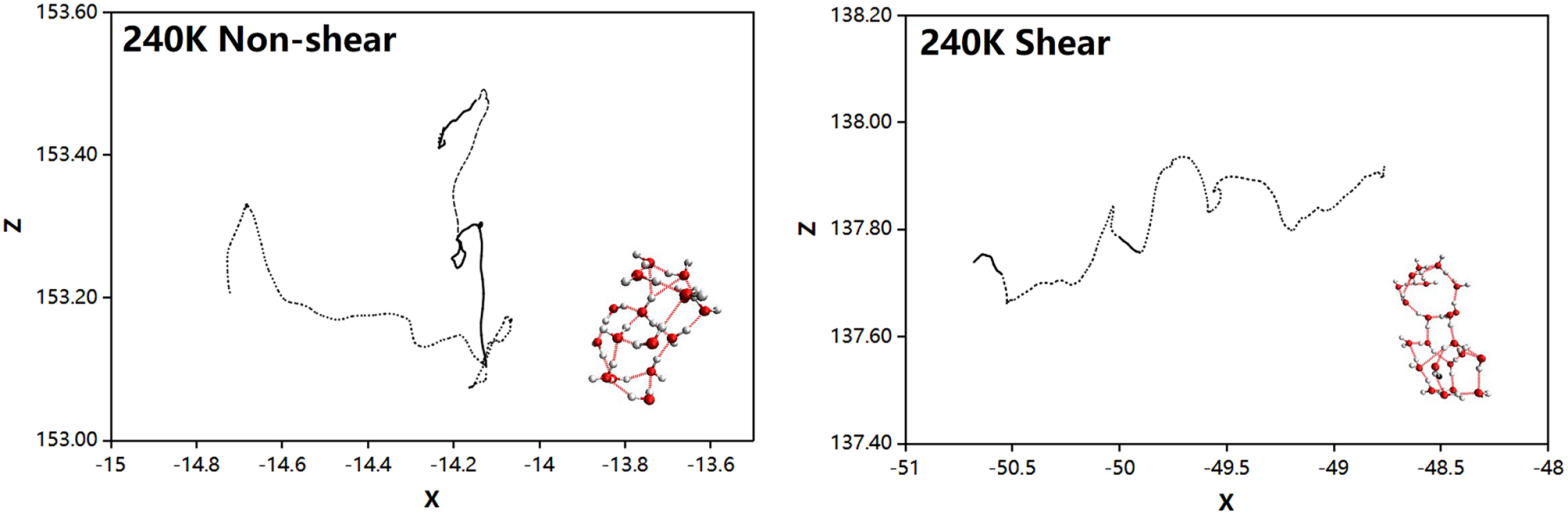
The projected trajectory of the centroid of the 4th- and 5th-level cluster masses at 240 K undergoing non-shear and shear. The solid line shows the trajectory during which the clusters exist. The dashed line shows the virtual trajectory during which the clusters are broken. Note that the scale factor for coordination and time is equivalent.

As shown in Fig. 6, in non-shear-driven flow, the trajectory is irregular and wanders around the starting point like Brownian movement. Under shearing, the center of mass of clusters has obvious translational movement along *X* direction, and the motions of *Z* direction are comparatively smaller.

### Structures of hierarchical clusters

The basic components of clusters are 4-, 5- and 6-membered rings, broadly distributed on the surface or interior of clusters. Referring to Matsumoto^31^, surface rings are defined as rings belonging to one fragment in clusters, whereas body rings are defined as rings belonging to more than one fragment. The rings that belong to the single-fragment clusters are classified as other types.

At the 4th level, the number of surface rings is approximately 3.3–3.6 times greater than that of body rings in various cases. The surface and body rings are gradually destroyed with heating or shearing. Compared with 4th-level, 5th-level clusters have more body rings and fewer surface rings.

At 240 K in the 4th-level clusters, the percentages of 4-, 5- and 6-membered surface rings in non-shear-driven flow are 10.54%, 22.67% and 66.79%, respectively. The percentages of body rings are 13.93%, 12.9% and 73.70%. All rings only including 4-, 5- and 6-membered rings in non-shear-driven flow are 8.83%, 37.73% and 53.44%. Compared with global system, the clusters tend to aggregate more 6-membered rings. The inner layer of clusters is mostly occupied by 6-membered rings. However, 5-membered rings are more favored in the surface layer compared with 4-membered rings. Kazimirski^36^ concluded that larger rings were concentrated preferentially in the inner part of the clusters, *n* = 48, 123 and 293. The reason for this is to strike a balance between tetrahedral coordination, which favors 6-membered rings, eliminating surface dangling atoms and increasing the number of hydrogen bonds, which favors 4-, 5-membered rings^36^. The conclusions are similar to our study. In addition, upon heating or shearing, both the interior and surface of clusters have more 4-, 5-membered rings whereas 6-membered rings decline. At 5th level, there are more 6-membered rings on the surface and more 4-, 5-membered rings inside the clusters. The detail is reported in Supplementary Table S5.

It has been explored that the origin of the anomalous behavior of liquid water could be due to two different components of a high-density liquid (HDL) and a low-density liquid (LDL)^18–20^. Camisasca^66^ proposed that clathratelike structures, similar to fused dodecahedra, can represent the LDL local structures as templates around which fluctuations occur, while chains can represent the HDL-like structures. The fused dodecahedra regarded as water clusters is mainly constituted by 4-, 5- and 6-membered rings, the distribution of which is similar to water clusters we proposed. Despite the fact that 5-membered rings are most favored by fused dodecahedra instead of 6-membered rings, both water clusters are ice-like clathrate structures and concrete manifests of density fluctuations. Interstitial water networks between water clusters are analogous to HDL, the structure of which is rather skewed and chain-like. Two different components can be searched out topologically, the results of which confirm spatial organization of two different local environments in water network.

## Conclusions and discussions

A hierarchical clustering method is firstly proposed to search out water clusters at all levels in hydrogen bond network based on graph community. The hierarchical clustering method not only shows good clustering performance but can also take the lifetime of clusters into consideration. Hydrogen bonds, rings and fragments are defined as 1st-, 2nd-, and 3rd-level structures, respectively. Higher-level clusters can be successively attained using modified Louvain algorithm based on the network of fragments.

Several types of fragments, including 5- and 6-membered rings, are favored. Approximately 25% of 5th-level clusters contain more than 20 water molecules and the maximum of 5th-level clusters size are more than 100 water molecules at low temperature. Based on 4th-level clusters, 5th-level clusters have distinct agglomerations of small clusters and a higher proportion of large clusters. More than 60% of clusters remain unchanged under our definition, and a small number of clusters, approximately 10–15%, are merged into other clusters or split into pieces. Temperature and shearing can both accelerate the disintegration of clusters and curtail the lifetime of clusters. In shear-driven flow, distinct cooperative translational motion of clusters is observed. For a cluster, the inner layer is mostly occupied by 6-membered rings, whereas 5-membered rings are more favored in the external surface layer than 4-membered rings. Upon heating or shearing, both the interior and surface of clusters form more 4-, 5-membered rings.

One concern is that the clusters are too flickering and unstable to explore the connections between the topology of clusters and the effect of shearing. Larger clusters may have longer life time because dynamics of hydrogen bonds mainly happen in the interior of clusters, which have less influence on the result of clustering. The stability of larger clusters in the micrometer-scale molecular dynamics simulation will be explored to confirm the surmise above and study detailed properties of large clusters.

Another concern is that the directions of hydrogen bonds are neglected for simplicity. The hierarchical clustering method will be optimized to introduce the directions of hydrogen bonds. Besides, the criterion of hydrogen bonds may have some impact on partitions. To test the sensitivity, we calculate water network under different distance cut-offs (0.345, 0.350, 0.355 nm). Compared with 0.35 nm cut-off, the percentage of identical clusters in 0.005 nm fluctuation of cut off is more than 85.0%, which indicates that the change of clustering is not in a binary fashion under different criterion. However, more accuracy methods to identify hydrogen bonds should be studied. At the same time, different water models may attain different partitions of water clusters. After the same simulation, TIP4P2005 have 3.0% more four-coordinated water molecules than SPC/E and in TIP4P/2005 water network there are obviously more clusters at both levels. In the future, to avoid the deviation from water models, machine-learning potential will be introduced to simulate liquid water for ab initio accuracy and faster efficiency of computation.

Hierarchical clustering method can attain large clusters successively. Fluid particles are equivalent to giant clusters under our definition. To form a bridge between macroscopic hydraulics and microscopic molecular dynamics, the method can be used to search for fluid particles in the micrometer-scale moleculer dynamics simulations which can apply to continuum mechanisms. Continuous liquid water can be discretized as an assembly of large clusters, as a result of which the properties of liquid water can be explained based on the characteristics of large clusters, especially viscosity and turbulence.

## Methods

### Model

**Figure 7:**
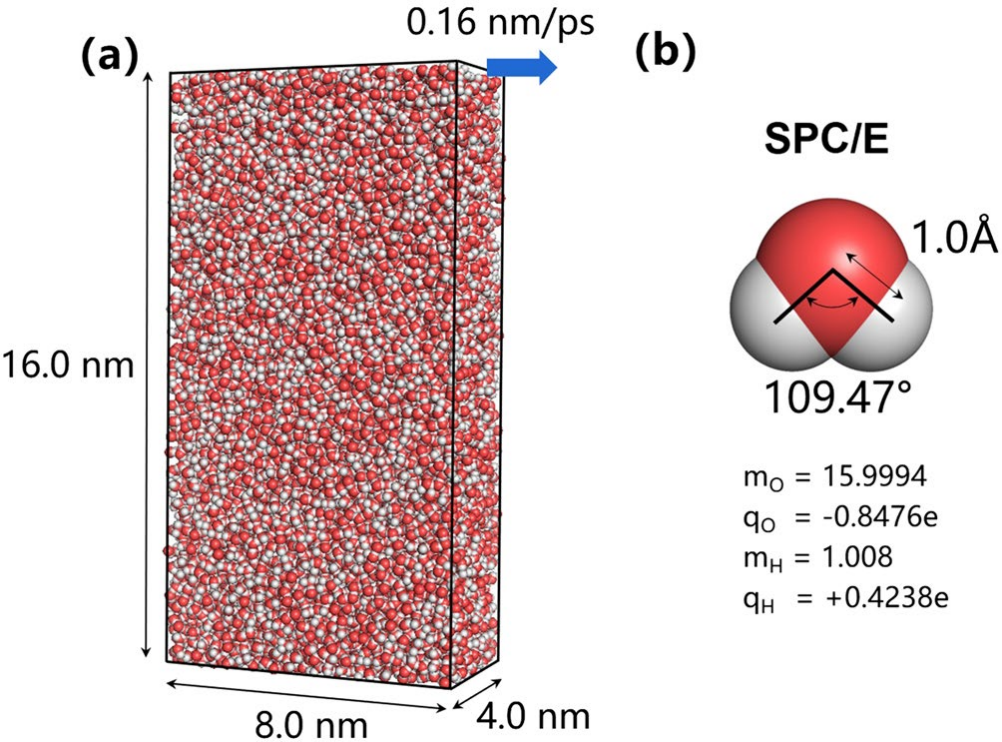
Schematics of the system model (**a**) and water model (**b**). (**a**) The system includes 17,314 water molecules. The shearing velocity is 0.16 nm/ps along x direction. (**b**) The geometry of SPC/E water model. The pictures are both drawn by VMD 1.9.1^63^.

We use LAMMPS software package to conduct molecular dynamics simulations. The system contains 17,314 water molecules of the extended simple point charge (SPC/E) water model^38^ in a box with periodic boundary conditions. The initial dimensions of the simulation box are 8.0 nm along the *x*-direction, 4.0 nm along the *y*-direction and 16.0 nm along the *z*-direction as shown in Fig. 7. The bond length and the bond angle of water are fixed with SHAKE algorithm. The PPPM method is used to calculate long-range Coulomb interaction. We use Lennard–Jones potential to describe the intermolecular interactions, given by

1 $$ U\left( r \right) = 4\varepsilon \left[ {\left( {\frac{\sigma }{r}} \right)^{12} - \left( {\frac{\sigma }{r}} \right)^{6} } \right],\quad r < r_{c} $$

where *ε*, *σ* are Lennard–Jones parameters, *r* is the distance between two atoms, *r*_*c*_ is the cutoff. As originally proposed, *ε*_O–O_ = 0.6492 kJ/mol, *ε*_H–H_ = 0.0 kJ/mol, *ε*_O–H_ = 0.0 kJ/mol, *σ*_O–O_ = 0.3166 nm, *σ*_H–H_ = 0.0 nm, *σ*_O–H_ = 0.0 nm, *r*_*c*_ = 1.1 nm.

Additionally, a Coulombic pairwise interaction within cut-off is given by

2 $$ Q\left( {r_{ij} } \right) = \frac{{Cq_{i} q_{j} }}{\zeta \cdot r},\quad r_{ij} < r_{c} $$

where *C* is an energy-conversion constant, *q*_*i*_ and *q*_*j*_ are the charges on the 2 atoms, and ζ is the dielectric constant. The charge of the oxygen atom *q*_*O*_ =  − 0.8476*e*, and the charge of the hydrogen atom *q*_*H*_ =  + 0.4238*e*. The Coulombic cut-off means that pairwise interactions within this distance are computed directly and interactions outside that distance are computed in reciprocal space with PPPM.

To study the effect of shearing, homogeneous non-equilibrium molecular dynamics (NEMD), called SLLOD, is used to simulate planar Couette flow. In this algorithm, fictitious mechanical forces are introduced to sustain shearing motion, and a homogeneous thermostat must be applied to attain a steady state^39^. The SLLOD equations can be written as

3 $$ \begin{aligned} \dot{r}_{i} & = \frac{{p_{i} }}{m} + r_{i} \cdot \nabla v \\ \dot{p}_{i} & = F_{i}^{\Phi } - p_{i} \cdot \nabla v \\ \end{aligned} $$

where *p*_*i*_ is mometum of atom *i*, *r*_*i*_ is displacement of atom *i*, *v*_*i*_ is velocity of atom *i*, *m* is mass of atom *i*, $F_{i}^{\Phi }$ is the interatomic force on atom *i* due to all other atoms. To construct shear-driven flow, the velocity of the top boundary is set to 0.16 nm/ps along x direction.

In this paper, we use SLLOD algorithm to simulate shear-driven flow compared with classical equilibrium MD for non-shear-driven flow. Considering that the melting temperature of SPC/E is 215 K^67^, All of the simulations are run at temperatures of 240 K, 260 K, 280 K, and 300 K and a pressure of 1.0 atm. A Nose–Hoover thermostat and barostat are used to control the temperature and pressure, respectively. The process of molecular dynamics simulations consists of three steps: the first step involves pre-equilibrium using an NVT ensemble for 2.0 ns. The second step is to ensure that the system attains a steady state using an NPT ensemble for 10.0 ns. Then, the third period of 7.0 ns is used to attain shearing flow using SLLOD algorithm. The time step is 1.0 fs. Finally, the last period outputs 500 configurations for a production run. MD trajectories including atomic coordinates are saved every 10 time steps for analysis.

### Hierarchical graph-based clustering method

Based on the network of fragments, a hierarchical clustering method is proposed to cut out water clusters from hydrogen bond network by successively merging lower-level clusters to form higher-level large clusters, taking *modularity* and *adjusted mutual information* into consideration.

#### Modularity

Modularity^68^ is a property of a network, describing the clustering efficiency of grouping a specific division of network into communities. High modularity means good clustering where there are many edges within communities and only a few between them.

The degree *k*_*i*_ of vertex *i* is defined as the number of edges connected with vertex *i*.

4 $$ k_{i} = \sum\limits_{j} {A_{ij} } $$

where *A*_*ij*_ is a parameter representing whether two vertices are adjacent. *A*_*ij*_ = 1 if two vertices are adjacent; otherwise, *A*_*ij*_ = 0.

The probability of an edge existing between vertices *i* and *j* if connections are made at random but respecting vertex degrees is *k*_*i*_* k*_*j*_/2 m.

The modularity *Q* is defined as

5 $$ Q = \frac{1}{2m}\sum\limits_{i,j} {\left( {A_{ij} - \frac{{k_{i} k_{j} }}{2m}} \right) \cdot \delta (c_{i} ,c_{j} )} $$

where the *δ*-function *δ*(*c*_*i*_, *c*_*j*_) equals 1 if *i* and *j* belong to the same cluster and otherwise, *c*_*i*_ denotes the cluster to which *i* belongs. If the network is randomized and homogeneous, *Q* is 0. A value above approximately 0.3 is a good indicator of significant community structure in a network^68^. A larger value means better clustering, and the maximum *Q* is 1.0.

#### Adjusted mutual information

Adjusted mutual information (AMI)^64^ is introduced to measure discrepancies in clustering results between neighboring configurations.

One assumes that *U*^*t*^ is the clustering result at time step *t* and *U*^*t*+1^ at time step *t* + 1. Their entropy is the amount of uncertainty for a partition set, as defined by:

6 $$ \begin{aligned} H(U^{t} ) & = - \sum\limits_{i = 1}^{{U^{t} }} {P(i)\log (P(i))} \\ H(U^{t + 1} ) & = - \sum\limits_{j = 1}^{{U^{t + 1} }} {P\prime (j)\log (P\prime (j))} \\ \end{aligned} $$

where *P*(*i*) is the probability that an object picked at random from *U* falls into class *U*_*i*_.

The probability is defined as

7 $$ \begin{aligned} P(i) & = \left| {U_{i}^{t} } \right|/N \\ P(j) & = \left| {U_{j}^{t + 1} } \right|/N \\ \end{aligned} $$

The mutual information (MI) between *U*^*t*^ and *U*^*t*+1^ is calculated using

8 $$ MI(U^{t} ,U^{t + 1} ) = \sum\limits_{i = 1}^{{\left| {U^{t} } \right|}} {\sum\limits_{j = 1}^{{\left| {U^{t + 1} } \right|}} {P(i,j)\log \left( {\frac{P(i,j)}{{P(i)P\prime (j)}}} \right)} } $$

Normalized against chance, AMI can then be calculated:

9 $$ AMI(U_{t} ,U_{t + 1} ) = \frac{MI - E[MI]}{{mean(H(U_{t} ),H(U_{t + 1} )) - E[MI]}} $$

AMI ranges from 0 to 1. If the value of AMI is close to zero, it indicates that two clustering results are largely independent. An AMI of exactly 1 indicates that two clustering results are equal.

#### Modified graph community

**Figure 8:**
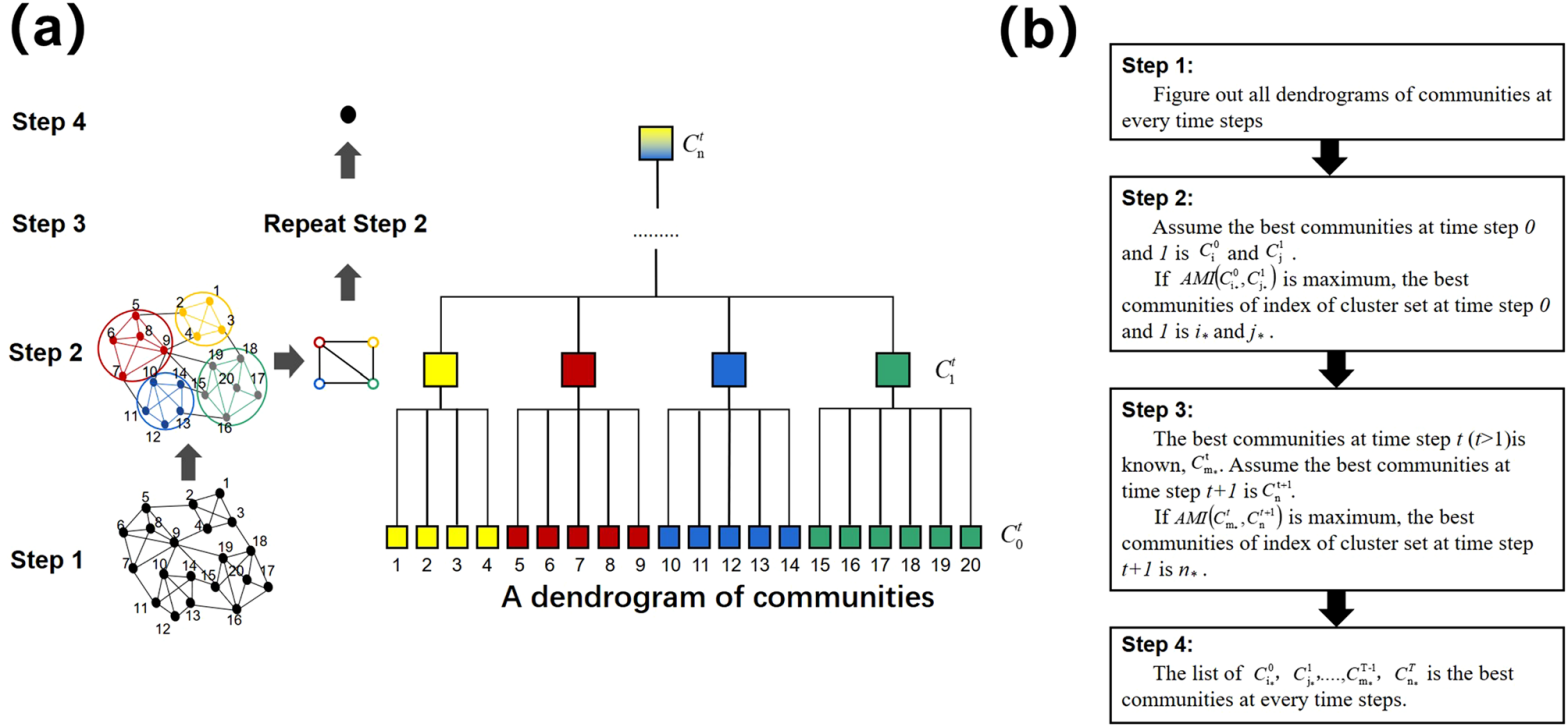
Schematics of the hierarchical clustering method. (**a**) Schematics of the Louvain algorithm. (**b**) Flowchart for the modified algorithm.

Traditionally, the graph community is used to determine the set of vertices that have denser connections with each other than other parts of the network^42,68–72^. The Louvain algorithm is a fast unfolding algorithm for computing hierarchical communities of a large network^42^. In Fig. 8a, the algorithm is divided into two parts repeated iteratively. First, we start with a weighted network of *N* nodes and the weight is the number of molecules shared by two structures. Step 1 regards every single node as an isolated community. Then, there are two parts in step 2. In the first part, we choose each node *i* and one of its neighbors *j*. The gain of modularity is evaluated after eliminating *i* and placing it into *j*. Node *i* is then placed in the community for which this gain is maximum only when this gain is positive. If this gain is negative, *i* stays in its original community. This process is repeated for all nodes until there is no further improvement in modularity. In the second part, we build a new network in which the nodes are the communities found in the first part. The weights of edges between new nodes are the sum of weights between every node in two communities. In step 3, we repeat the operation of step 2 to obtain a dendrogram of clustering for the network. This dendrogram represents a hierarchical decomposition of network into communities at all levels. In the dendrogram, *C*_*i*_^*t*^ means a set of clusters, where *i* is the level of clustering and *t* is the time step of the configuration. Ultimately, maximum modularity corresponds to the best graph community.

However, the dynamics of the best graph community have almost poor stability performance. The dynamics of hydrogen bonds result in great changes in the best community between small intervals. To gain a set of comparatively stable clusters, we propose a modified algorithm for the graph community that takes the parameters of measured clustering stability and *adjusted mutual information* into consideration. In Fig. 8b, the process of the modified algorithm is illustrated. One assumes that there are *T* configurations in chronological order. In step 1, we should determine all the dendrograms for the communities in every configuration. In step 2, we ascertain the best communities for the first two configurations. We assume the best communities at time steps 0 and 1 are *C*_*i*_^0^ and *C*_*j*_^1^, respectively. If *AMI*(*C*_i*_^0^, *C*_j*_^1^) reaches the maximum, the best communities of index for the cluster set at time steps 0 and 1 are i* and j*. In step 3, on the condition that the best communities *C*_*m*_^*t*^ at time step *t* (*t* > 1) are known, we assume that the best communities at time step *t* + 1 are *C*_*n*_^*t*+1^. If *AMI*(*C*_m*_^t^, *C*_n*_^t+1^) is at its maximum, the best community of the index of the cluster set at time step *t* + 1 is n*. We repeat step 3 iteratively, and, in the end, we regard the list of *C*_i*_^0^, *C*_j*_^1^…, *C*_m*_^T−1^, *C*_n*_^T^ as the best communities in every configuration.

#### Hierarchical clustering

This hierarchy of clusters is represented as a tree. The root of the tree is composed of the 3rd-level clusters, fragments. The process of hierarchical clustering involves two parts. First, we should construct the network of the structures at the present level. If two structures have shared molecules, there exists an edge between two nodes, the weights of which are the number of shared molecules. Secondly, based on the network, we can obtain a list of structures at higher level by using modified Louvain algorithm. The criteria to exit the hierarchical clustering is as follows. Assume that the numbers of *N*th-level and *N* + 1th-level cluster are *n*_*t*_^*N*^ and *n*_*t*_^*N*+1^ at time step *t*. The clustering algorithm will be stopped if for all time steps t, *n*_*t*_^*N*^ = *n*_*t*_^*N*+1^ or |*n*_*t*_^*N*^ − *n*_*t*_^*N*+1^*|*/*n*_*t*_^*N*^ < 0.1% (when *n*_*t*_^*N*^ = *n*_*t*_^*N*+1^ can not achieve).

## Supplementary information

Supplementary Informations.
