# Supplementary material for: A hierarchical clustering method of hydrogen bond networks in liquid water undergoing shear flow
Source: Sci Rep. 2021 May 5;11:9542. doi: 10.1038/s41598-021-88810-7 (PMC8100111; doi:10.1038/s41598-021-88810-7)
Supplement: Supplementary file 1 — Supplementary Informations. [file 41598_2021_88810_MOESM1_ESM.pdf]

Supplemental materials for "A hierarchical clustering method of hydrogen bond networks in liquid water undergoing shear flow"

Yitian Gao, Hongwei Fang, Ke Ni

State Key Laboratory of Hydro-science and Engineering, Department of Hydraulic Engineering, Tsinghua University, Beijing 100084, China

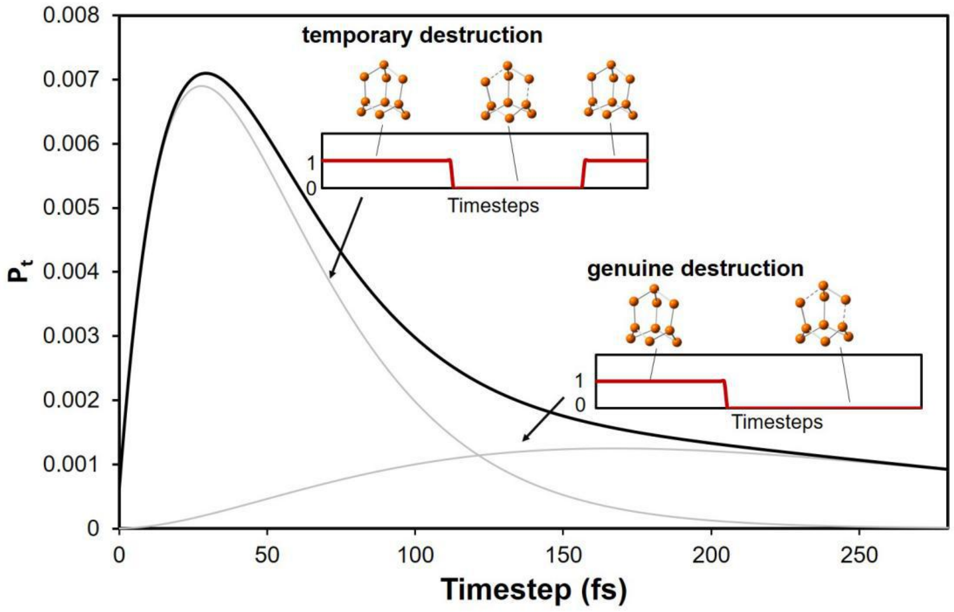

Figure S1. The distribution of the total lifetime of fragments in the instantaneous configuration considered. The black line shows the total lifetime distribution. The gray lines are two exponents in our approximation.

The arbitrary cutoff distance for hydrogen bonds leads to transient breaking and the formation of hydrogen bonds more frequently[1,2,3]. Voloshin[1] concluded that the distribution of transient hydrogen bonds and transient breaking of hydrogen bonds reach maxima at ~15.0 fs and ~130.0 fs, respectively. It is assumed that the short-term bond breaking of less than 1 ps can be regarded as a part of its total lifetime instead of genuine breaking[1]. Neglecting transient breaking may reproduce better results for the hydrogen bond lifetime with experiments[3]. Similar to the definition above, a correlation function can be introduced to describe the probability that the fragment is intact at time *t*, given that it is intact at time 0:

$$C_a^{fragment}(t) = \frac{\langle h(0)h(t)\rangle}{\langle h\rangle} \tag{1}$$

where $h(t)$ is 1 when the fragment is intact at time t and 0 otherwise.

The average rate of change of the fragment population $P_d^{fragment}(t)$ is given by

$$P_a^{fragment}(t) = -\frac{dC_a^{fragment}(t)}{dt} \tag{2}$$

The distribution $P_c^{fragment}(t)$ is expressed as

$$P_c^{fragment}(t) = -t\frac{dP_a^{fragment}(t)}{dt} \tag{3}$$

The average lifetime of rings can be defined as

$$\left\langle t_c^{cluster}\right\rangle = \int_0^\infty t\cdot P_c^{cluster}(t)dt \tag{4}$$

In Fig. S1, similar to the approximation of hydrogen bonds, the distribution of fragments can be approximated by the function

$$P_c(t) = 0.01875\left[\frac{t}{28.80}e^{-\frac{t}{28.80}} + 0.1230\left(\frac{t}{84.14}\right)^2 e^{-\frac{t}{84.18}}\right] \tag{5}$$

A correlation function can be also introduced to describe the probability that a cluster is intact at time *t*, given that it is intact at time *0*:

$$C_a^{\mathrm{cluster}}(t) = \frac{\langle h(0)h(t)\rangle}{\langle h\rangle} \tag{6}$$

$$C_a^{\mathrm{cluster}}(t) = \frac{\langle h(0)h(t)\rangle}{\langle h\rangle}$$

where *h(t)* is 1 when the cluster is intact at time t and 0 otherwise.

The average rate of change of cluster population $P_a^{cluster}(t)$ is given by

$$P_a^{cluster}(t) = -\frac{dC_a^{cluster}(t)}{dt} \tag{7}$$

$$P_a^{cluster}(t) = -\frac{dC_a^{cluster}(t)}{dt}$$

The distribution $P_c^{cluster}(t)$ is expressed as

$$P_c^{cluster}(t) = -t\frac{dP_a^{cluster}(t)}{dt} \tag{8}$$

$$P_c^{cluster}(t) = -t\frac{dP_a^{cluster}(t)}{dt}$$

The average lifetime of clusters can be defined as

$$\left\langle t_c^{cluster}\right\rangle = \int_0^{\infty} t \cdot P_c^{cluster}(t)dt \tag{9}$$

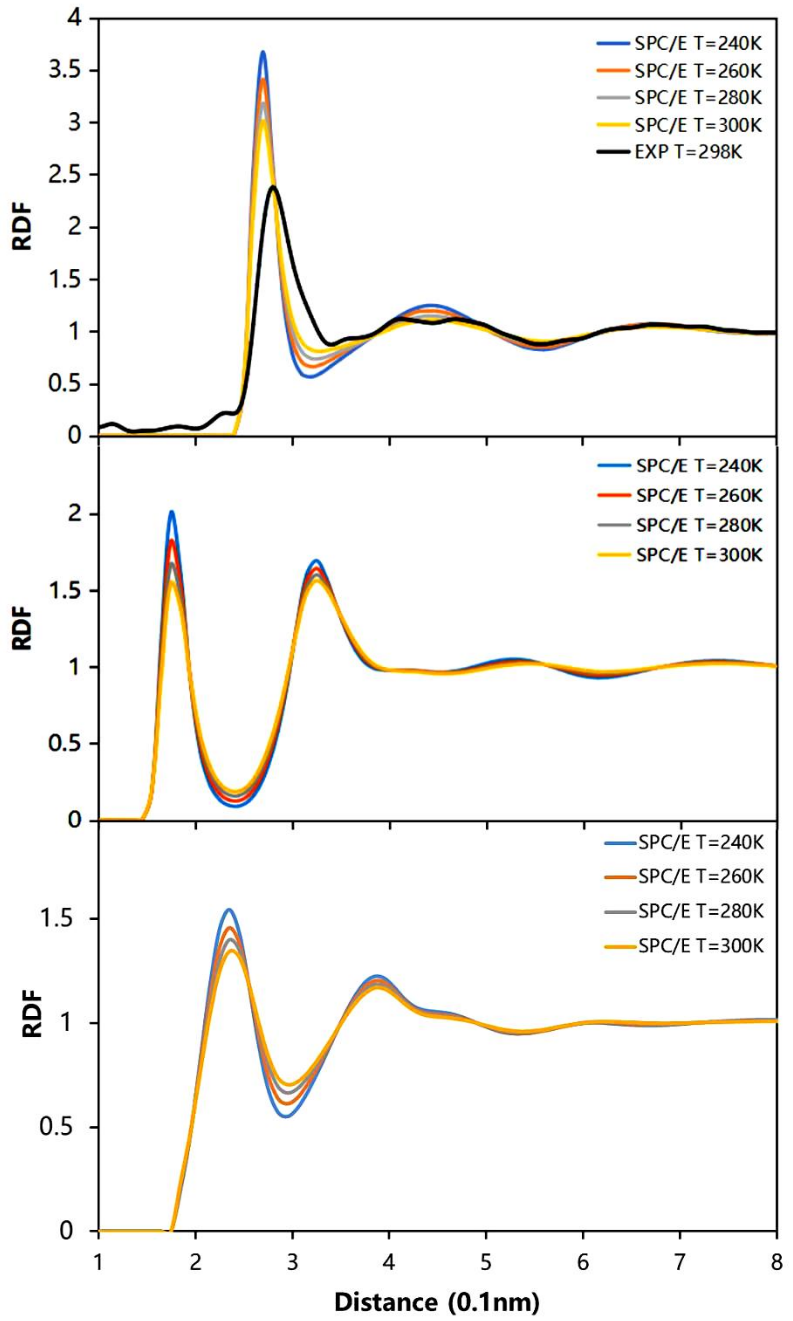

Figure S2. The radial distribution function (RDF) of oxygen-oxygen, hydrogen-oxygen and hydrogen-hydrogen at various temperatures.

Table S1. The density and viscosity determined from the experiment and simulations.

Temperature(K)	Density (g/cm^3)			Viscosity (mPa.s)		
	Expt.	Molecular Dynamics		Expt.	Molecular Dynamics	
		SPC/E	Ref.		SPC/E	REF.
240		1.013	1.012 (240 K)[7]		1.280	
260		1.011	1.010 (260 K)[7]		1.080	
280	1.000	1.007	1.008 (273 K)[7]	1.428	0.923	1.010 (283 K)[5]
300	0.998	0.997	0.994 (300 K)[5]<br>0.999 (303 K)[6]	0.855	0.772	0.729 (298 K)[4]<br>0.722 (300 K)[5]<br>0.65 (303 K)[6]

Table S2. The AMI value for hierarchical structures at different levels in the network in various cases.

T (K)	4th-level		5th-level	
	Non-shear	Shear	Non-shear	Shear
240	93.41%	91.38%	93.39%	91.37%
260	91.05%	90.25%	91.03%	90.24%
280	88.89%	88.76%	88.88%	88.75%
300	86.62%	87.66%	86.62%	87.66%

Table S3. The proportion of 4th- and 5th-level clusters under different patterns of change in various cases.

		**4th-level clusters**				**5th-level clusters**			
		240 K	260 K	280 K	300 K	240 K	260 K	280 K	300 K
(1)	non-shear	76.61%	72.05%	69.00%	66.80%	73.27%	69.17%	67.23%	64.77%
	shear	74.08%	71.68%	70.43%	69.45%	72.38%	70.16%	69.22%	68.52%
(2)	non-shear	9.80%	11.13%	11.83%	12.28%	11.64%	12.91%	13.17%	13.47%
	shear	10.30%	11.02%	11.04%	11.06%	11.64%	11.80%	12.15%	12.01%
(3)	non-shear	9.60%	10.90%	11.57%	12.01%	11.28%	12.52%	12.77%	13.12%
	shear	10.13%	10.79%	10.94%	10.87%	11.36%	3.16%	11.90%	11.76%
(4)	non-shear	1.67%	2.05%	2.20%	2.20%	3.59%	3.95%	3.77%	3.38%
	shear	1.68%	1.89%	1.79%	1.74%	2.86%	3.16%	2.76%	2.50%

Table S4. The different types of average lifetimes of the 4th- and 5th-level clusters at 240 K. t_1 denotes the average lifetime of all clusters. t_2 denotes the average lifetime of the clusters, the size of which is more than 1 fragment. t_3 denotes the average lifetime of large clusters, the size of which is more than 5 fragments.

	non-shear driven flow		shear driven flow	
	4th level	5th level	4th level	5th level
t_1	150.60	158.07	131.69	135.25
t_2	110.75	93.91	91.43	80.40
t_3	59.63	44.75	52.10	40.27

Table S5. The proportional distribution of different types of rings in the 4th- and 5th-level clusters in various cases.

		**Surface rings**			**Body rings**		
		4	5	6	4	5	6
240 K	non-shear	10.54%	22.67%	66.79%	13.93%	12.37%	73.70%
	shear	15.85%	24.15%	60.00%	18.08%	16.15%	65.77%
260 K	non-shear	12.93%	22.64%	64.43%	15.74%	12.50%	71.76%
	shear	17.04%	24.71%	58.25%	18.84%	16.81%	64.35%
280 K	non-shear	14.96%	23.65%	61.39%	18.05%	14.75%	67.20%
	shear	17.67%	25.36%	56.97%	20.15%	18.24%	61.61%
300 K	non-shear	16.09%	24.13%	59.78%	18.99%	15.57%	65.44%
	shear	18.99%	26.42%	54.59%	21.56%	19.95%	58.49%

References

1. Galamba, N. On the Effects of Temperature, Pressure, and Dissolved Salts on the Hydrogen-Bond Network of Water. The Journal of Physical Chemistry B 117, 589-601 (2013).
2. Belosludov, V. et al. The nano-structural inhomogeneity of dynamic hydrogen bond network of TIP4P/2005 water. Scientific Reports 10 (2020).
3. Clauset, A., Newman, M.E.J. & Moore, C. Finding community structure in very large networks. Physical review. E, Statistical, nonlinear, and soft matter physics 70, 066111-066111 (2004).

4. González, M.A. & Abascal, J.L.F. The shear viscosity of rigid water models. The Journal of Chemical Physics 132, 096101 (2010).
5. Song, Y. & Dai, L.L. The shear viscosities of common water models by non-equilibrium molecular dynamics simulations. Molecular simulation 36, 560-567 (2010).
6. GUO, G. & ZHANG, Y. Equilibrium molecular dynamics calculation of the bulk viscosity of liquid water. Molecular physics 99, 283-289 (2001).
7. Bryk, T. & Haymet, A.D.J. The Ice/Water Interface: Density-Temperature Phase Diagram for the SPC/E Model of Liquid Water. Molecular simulation 30, 131-135 (2004).
